# Skin models for cutaneous melioidosis reveal *Burkholderia* infection dynamics at wound’s edge with inflammasome activation, keratinocyte extrusion and epidermal detachment

**DOI:** 10.1080/22221751.2021.2011621

**Published:** 2021-12-06

**Authors:** Joanne Wei Kay Ku, Supatra Tharinee Marsh, Mui Hoon Nai, Kim Samirah Robinson, Daniel Eng Thiam Teo, Franklin Lei Zhong, Katherine A. Brown, Thiam Chye Lim, Chwee Teck Lim, Yunn-Hwen Gan

**Affiliations:** a Department of Biochemistry, National University of Singapore, Singapore, Singapore; b Department of Biomedical Engineering, National University of Singapore, Singapore, Singapore; c Skin Research Institute of Singapore (SRIS), Immunos, Singapore; d Lee Kong Chian School of Medicine, Nanyang Technological University, Singapore, Singapore; e Cavendish Laboratory, University of Cambridge, Cambridge, UK; f Oden Institute for Computational Engineering and Sciences, The University of Texas at Austin, Austin, TX, USA; g Division of Plastic, Reconstructive &Aesthetic Surgery, National University Health System, Singapore, Singapore; h Institute for Health Innovation and Technology (iHealthtech), National University of Singapore, Singapore, Singapore; i Infectious Diseases Translational Research Program, Yong Loo Lin School of Medicine, National University of Singapore, Singapore, Singapore

**Keywords:** Burkholderia, inflammasome, keratinocyte, melioidosis, skin

## Abstract

Melioidosis is a serious infectious disease endemic in Southeast Asia, Northern Australia and has been increasingly reported in other tropical and subtropical regions in the world. Percutaneous inoculation through cuts and wounds on the skin is one of the major modes of natural transmission. Despite cuts in skin being a major route of entry, very little is known about how the causative bacterium *Burkholderia pseudomallei* initiates an infection at the skin and the disease manifestation at the skin known as cutaneous melioidosis. One key issue is the lack of suitable and relevant infection models. Employing an *in vitro* 2D keratinocyte cell culture, a 3D skin equivalent fibroblast-keratinocyte co-culture and *ex vivo* organ culture from human skin, we developed infection models utilizing surrogate model organism *Burkholderia thailandensis* to investigate *Burkholderia*-skin interactions. Collectively, these models show that the bacterial infection was largely limited at the wound’s edge. Infection impedes wound closure, triggers inflammasome activation and cellular extrusion in the keratinocytes as a potential way to control bacterial infectious load at the skin. However, extensive infection over time could result in the epidermal layer being sloughed off, potentially contributing to formation of skin lesions.

## Introduction

Melioidosis is a potentially fatal infectious disease endemic in the tropics [1,2] with an estimated fatality of 89,000 persons per year worldwide [1]. Disease manifestations vary from localized cutaneous infection such as skin ulcers or soft tissue abscesses to disseminated disease and can even lead to disseminated disease, and potentially fatal sepsis [1,2]. The causative agent, *Burkholderia pseudomallei* (Bp), is an environmental saprophyte found in soils and surface groundwater [3,4].

Natural infection by Bp can occur via percutaneous inoculation, inhalation or ingestion of contaminated soils or water [1,2]. Percutaneous inoculation of skin cuts and abrasions are considered a major mode of Bp infection, with melioidosis commonly reported in individuals with open wounds and rice farmers in frequent contact with contaminated water and soil [5–8]. About 25% of melioidosis patients recalled having inoculating skin injuries prior to the onset of melioidosis in a retrospective study conducted in Australia [9].

Cutaneous melioidosis is a rare infection that presents as nonspecific abscess or ulceration, especially in residents of, or returned travellers from endemic countries [10]. In Australia, cutaneous melioidosis accounts for 12% of melioidosis cases, and is classified as either a primary or disseminated secondary skin infection [11]. The majority of primary skin melioidosis patients had non-specific single lesions, and the most common presentation was ulcer with or without purulent exudate.

Skin is the largest organ in the body, and even the smallest breaks that may be invisible to the naked eye are potential portals of entry for pathogenic microbes. Yet infection processes at the skin are poorly studied, contributed by a lack of suitable and accessible models. Very few studies have examined the pathogenesis of Bp via percutaneous inoculation in an animal model [12] or *in vitro* infection with keratinocytes [13].

Therefore, using *in vitro* 2D keratinocyte cell culture, 3D skin equivalent fibroblast-keratinocyte co-culture and *ex vivo* organ culture (EVOC) from human skin, we developed models of cutaneous infection utilizing *B. thailandensis* (Bt) as a surrogate to investigate Burkholderia-skin interactions. Bt is a relatively non-pathogenic close relative of Bp. The two organisms occupy similar ecological niches and overlap in their geographical distribution [14]. Bt also shares a similar intracellular lifecycle [15] and possesses homologs of Bp virulence factors [16,17]. Thus, Bt is commonly used as a surrogate organism to study Bp virulence mechanisms and pathogenesis as the latter is a risk group 3 organism and potential bioterrorism agent [18–20]. This report documents the infection dynamics of *Burkholderia* at the wounded skin site and provides the first evidence of the role of inflammasomes and cellular extrusion in the keratinocytes as a potential way to control bacterial infectious load at the skin. However, extensive infection over time could result in the epidermal layer being sloughed off, potentially contributing to formation of skin lesions such as ulcers.

## Materials and methods

### Cell culture

Primary human neonatal fibroblasts (Lonza) were grown in Dulbecco’s Modified Eagle Medium (DMEM) supplemented with 10% FBS (fetal bovine serum) and 1% penicillin–streptomycin (PS). Primary human neonatal keratinocytes (Lonza) and N/TERT-1 keratinocyte cell line (gift from Prof. Birgit Lane) were cultured in keratinocyte growth medium supplemented with hydrocortisone, transferrin, epinephrine, GA-1000, bovine pituitary extract, human epidermal growth factor, and insulin (KGM Gold Keratinocyte Growth Medium BulletKit, Lonza). N/TERT-1 with stable expression of GFP-LifeAct, GFP-ASC, or CRISPR-Cas9 edited genetic knockouts: ASC, CASP1 or GSDMD KO cells were periodically selected with puromycin (2 μg/mL). Cells were cultured at 5% CO_2_, 37 °C in a humidified incubator. The absence of mycoplasma contamination was confirmed using MycoAlertTM PLUS Mycoplasma Detection Kit (Lonza).

### 3D skin equivalent culture

To construct the fibroblast populated fibrin gel, primary human neonatal fibroblasts were trypsinized and resuspended at a concentration of 4 × 10^5^ cells/mL in 3 U/mL thrombin (Sigma). The cell suspension was then mixed at a 1:1 ratio with 35 mg/mL fibrinogen (Sigma), giving a final concentration of 2 × 10^5^ cells/mL. Transwell 24-well inserts with 0.4 µm Pore Polyester Membrane (Corning) were used to polymerize 100 μL of fibrin-fibroblast gel mix at room temperature for 20 min. The resulting fibrin gel was submerged in DMEM with 10% FBS and 1% PS overnight. N/TERT-1 keratinocytes with stable expression of GFP-LifeAct was seeded on top of the fibrin gel (1 × 10^5^ cells/gel) in KGM Gold medium and cultured for 2 days before switching to Keratinocyte Differentiation Medium (KDM; Atlantis Bioscience) for 1 day. The cultures were then exposed to air on the top with 300 μL KDM replaced daily underneath the insert and cultured for 14 days at air/liquid interface. All media used for skin-on-chip culture was supplemented with 10 μg/mL aprotinin (Sigma) to inhibit enzymatic fibrinolysis of the gel during culture. After 14 days, culture skin equivalents were wounded using a 27-gauge needle before infection with Bt at MOI 100.

### EVOC

The study was conducted with approval by the Ethics Committee of the National University Hospital (NUH), (Singapore DSRB 2019/00092), and with the full informed consent of the patients. Eight female patients with ages ranging from 40 to 60 years, were included in the study.

Human abdominal skin – with subcutaneous fat layer removed – were obtained from routine cosmetic and reconstructive surgical procedures. Skin was washed thrice in 1X phosphate-buffered saline (PBS) and sterilized in KDM supplemented with 300 U/mL penicillin, 300 µg/mL streptomycin, and 0.75 µg/mL Gibco amphotericin B for 1 h (h) at 4°C. After sterilization, skin was washed thrice in PBS and wounded by cutting with a scalpel through the epidermis but not the dermis, so the skin was still intact. An 8 mm punch biopsy was used to cut out disks of skin with the scalpel cut localized to the middle of the tissue. The skin was then placed into 24-well transwell inserts with 0.4 µm pore polyester membrane. Air–liquid interface EVOCs were incubated overnight at 5% CO_2_, 37°C with KDM underneath the insert and the top of skin exposed to air, before bacterial infection the following day.

### Infection of skin models

*B. thailandensis* strain E264 *tssA::mApple*, a soil isolate obtained from Central Thailand expressing mApple fluorescent protein chromosomally, was used and routinely maintained on lysogeny broth agar [21].

Bt *tssA::mApple* was opsonized with 50% human serum (Sigma, H4522) for 1 h at 37 °C, 150 rpm prior to infection. Paraformaldehyde (PFA; Sigma) fixed Bt *tssA::mApple* was used as a control for assays and was generated by treating Bt *tssA::mApple* with 4% PFA at room temperature for 15 min. Bacteria were washed twice with 10 mM glycine in 1X PBS to remove traces of PFA and resuspended in 50% human serum.

Cell densities for 3D skin equivalent and EVOCs were estimated based on the average keratinocyte cell size of about 300 µm^2^ at low passages [22].

Cells were then infected with opsonized Bt strains at the indicated multiplicity of infections (MOIs) and centrifuged at 250*g* for 5 min at room temperature to ensure maximum bacteria-to-cell contact. 1-hour post-infection (hpi), media was replaced to KDM containing 1% PS and 300 µg/mL kanamycin to kill off extracellular bacteria.

### ELISA

Culture supernatants of Bt infected cells were obtained for determining cytokine production. IL-1β production was assessed via LEGEND MAX kit (Biolegend) according to manufacturer’s instructions.

### Live confocal imaging of infected 2D keratinocyte cell cultures

N-TERT-1 keratinocytes carrying the ASC-GFP reporter were infected with mApple fluorescent Bt at MOI 200. Prior to imaging, the media was replaced to KDM containing 1% PS, 300 µg/mL kanamycin and various dyes. 2 µM cell-impermeant nucleic acid stain, TO-PRO-3, was used to monitor cell death. 1:10,000 diluted CellMask Deep Red Plasma membrane stain was used to label plasma membrane. Cells were visualized on a spinning disk system based on an inverted microscope (Ti-E; Nikon), a spinning disk scan head (CSU-W1; Yokogawa), a sCMOS camera (Prime95B; Teledyne Photometrics), and a laser system (iLaunch; GATACA Systems). Samples were imaged with 20X NA 0.75 Plan Apo and 40X NA 1.15 Plan Apo objectives under laser excitation 488/561 nm and scanned with *Z*-series at step size 0.5 μm. Images were acquired at 30-minute intervals.

### Live cell imaging of in vitro scratch assay to visualize cell migration

N-TERT-1 keratinocytes with stable expression of LifeAct-GFP were grown to confluency for scratch assays. A gap was created by scratching a confluent cell monolayer with a P200 tip. Cells were infected with Bt *tssA::mApple* at the indicated MOI or treated with equal amounts of PFA-fixed Bt *tssA::mApple* as described before. Cells were then visualized using LSM710 (Zeiss) at a magnification of 20X at intervals of 20 min.

### Quantification of gap closure

A gap was generated via the seeding of N/TERT-1 keratinocytes in 35 mm dishes containing 2-well culture inserts (ibidi) or via scratching with a P200 tip. For visualization of the cells, CellMask Deep Red Plasma membrane stain or N-TERT-1 keratinocytes with stable expression of LifeAct-GFP were utilized. Cells were infected with Bt *tssA::mApple* at the indicated MOI or treated with equal amounts of PFA-fixed Bt *tssA::mApple* as described before. At 24 hpi, cells were visualized using LSM710 (Zeiss) or Nikon Ti2 motorized inverted microscope, X-Xite Xylis LED light source, at an objective magnification of 10X to obtain the size of the gap. The gap area was quantified using FIJI by manually drawing around the area. Data were expressed as fold-change from uninfected controls to normalize all three datasets.

### Quantitation of extruded cell via counting

N-TERT-1 keratinocytes and the corresponding ASC and CASP1 KO cells were infected at MOI 200 as described above. At 48 hpi, cell supernatants containing extruded cells were fixed with 4% PFA at room temperature for 15 min. Cells were then spun down at 15,000 g for 3 min and resuspended in 1X PBS. Cell were stained with 0.2% trypan blue and counted with a standard hemocytometer. The derived cell concentrations were used to determine total extruded cell counts.

### Assessing bacterial loads via image analysis

N-TERT keratinocytes and the corresponding ASC, CASP1 and GSDMD KO cells were infected at MOI 200 as described above. At 24 hpi, cells were fixed for 15 min in 4% PFA. 1:10,000 diluted CellMask Deep Red Plasma membrane stain was used to label plasma membrane while nuclei were stained with 10 µg/mL Hoechst 33342. Confocal scan slide images corresponding to an area of 3.57 × 3.57 mm^2^ were acquired using a spinning disk system based on an inverted microscope (Ti-E; Nikon), a spinning disk scan head (CSU-W1; Yokogawa), a sCMOS camera (Prime95B; Teledyne Photometrics), and a laser system (iLaunch; GATACA Systems). Samples were imaged with 20X NA 0.75 Plan Apo objectives under laser excitation 405/561/642 nm and scanned with Z-series at step size 1 μm. Extracellular bacteria were excluded via Imaris 3D surface rendering using Cell Mask signals to define intracellular regions. Images were analyzed using FIJI to threshold the red channel (visualization of extracellular Bt *tssA::mApple*) and produce a binary image to count the number of infection events over the 3.57 × 3.57 mm^2^ imaging area.

### H&E, immunostaining of EVOCs and confocal microscopy

EVOCs were embedded in Tissue-Tek O.C.T. 4583 compound, snap-frozen at 6, 24, 48 and 72 hpi and stored at −80°C. Serial cryostat sections (30 μm in thickness) were placed on glass slides, air-dried and fixed in 4% PFA in 1X PBS. Sections were then processed for staining with hematoxylin and eosin (H&E) or for immunofluorescent analysis as noted below.

EVOCs fixed at 6 and 24 hpi were permeabilized with 0.1% Triton-X prior to staining with phalloidin conjugated to AF488 and Hoechst 33342. EVOCs fixed at 48 and 72 hpi were non-permeabilized, blocked (2% BSA in 1X PBS) and incubated with the primary antibody (C6805 monoclonal mouse anti-collagen type VII antibody (Sigma-Aldrich)), Ab155233 rabbit polyclonal anti-gasdermin D (abcam) in blocking buffer), secondary antibody (A21052 AF633 goat anti-mouse antibody (Invitrogen), A11008 AF488 goat anti-rabbit antibody (Invitrogen) in blocking buffer) and the nuclei were stained with Hoechst 33342. The slides were mounted using Prolong Glass anti-fade (Invitrogen).

The sections were stained using the same protocol with the replacement of anti-Gasdermin D with PA1-41004 Histone H2A.X antibody (Invitrogen) as an isotype control antibody against an intracellular protein H2A.X.

Sections were imaged using a spinning disk system based on an inverted microscope (Ti-E; Nikon), a spinning disk scan head (CSU-W1; Yokogawa), a sCMOS camera (Prime95B; Teledyne Photometrics), and a laser system (iLaunch; GATACA Systems). The sections were imaged with 20X NA 0.75 Plan Apo and 100X NA 1.45 Plan Apo oil objectives under laser excitation 405/488/561/640 nm, DIC and scanned with Z-series μm at step size 0.5.

### Statistical analysis

Unpaired two-tailed t-test was used to determine the statistical significance of 95% confidence between two groups. One-way ANOVA followed by Tukey’s test was conducted to determine the statistical significance of 95% confidence between three groups or more. GraphPad Prism software was used for the calculation of statistics. Statistical significance is indicated as follows: *p ≤ 0.05; **p ≤ 0.01; ***p ≤ 0.001.

## Results

### *B. thailandensis* infection of the wounded skin

**Figure 1.**
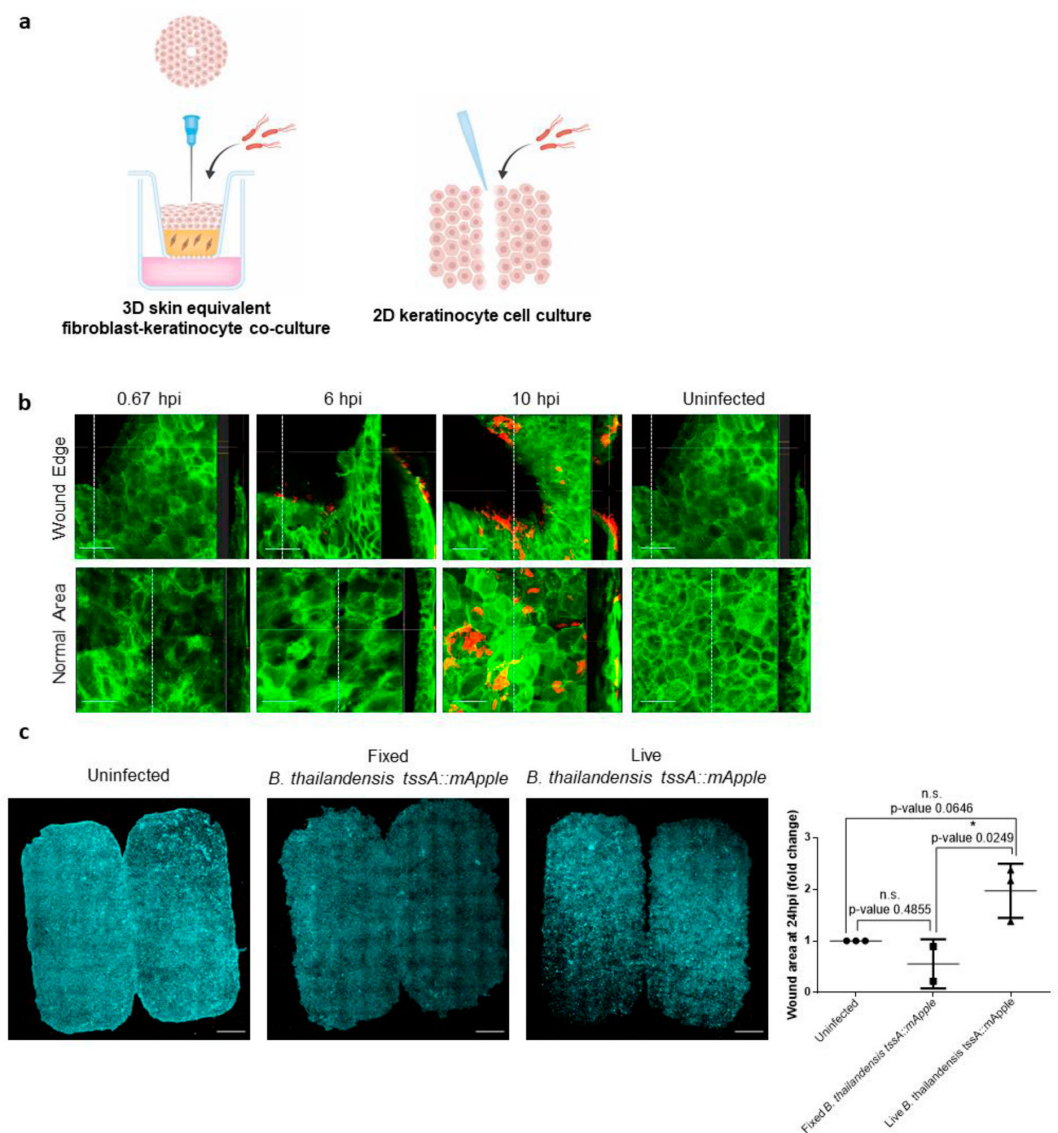
Bt infection of *in vitro* wound skin models. (a). Schematic of 3D skin equivalent and 2D scratch wound assay. (b). 3D skin equivalent culture of GFP LifeAct expressing N/TERT-1 keratinocytes wounded with a needle and cultured with Bt*,* fixed at 0.67, 6 and 10 hpi. Uninfected cultures fixed at 10 hpi was used as a control. *Z*-stack images with cross-sectional views (at white dotted vertical lines) of the wound edge and an undamaged/normal area in the same culture are displayed. Scale bar = 50 µm. (c). 2D scratch wound assay of N/TERT-1 keratinocytes infected with live Bt at 24 hpi. Uninfected cultures and PFA-fixed Bt were used as controls. Cells were stained with Cell Mask, displayed in cyan. Scale = 1 mm. Quantified wound area is expressed as fold-change relative to the uninfected control. Data presented as mean ± S.D from at least 2 independent experiments.

A 3D skin equivalent model was generated by culturing human fibroblasts in a fibrin hydrogel with human keratinocytes cultured on the apical side of the gel and grown at an air/liquid interface to induce epidermal differentiation. Once a stratified epidermis has formed, a 27-gauge needle was used to puncture the skin barrier causing a circular wound before inoculation with Bt expressing red fluorescence (Figure 1(a))*.* Cultures were imaged by confocal microscopy to acquire 3D rendered imaging of the wound after 40 min (S1 Movie), 6 h (S2 Movie), and 10 h (S3 Movie), with uninfected cultures used as a control (S4 Movie). Infected keratinocytes were localized around the wound edge with progressive accumulation of infected cells at later timepoints at 6 and 10 h (bacteria in red) (Figure 1(b)). Bacteria were also seen to infect undamaged areas of the skin equivalent at 10 h (Figure 1(b); normal area). However, this was localized to the top layer of keratinocytes, suggesting bacteria were not able to penetrate into the deeper layers.

We also performed experiments in the 2D keratinocyte culture model (Figure 1(a)) that allows for live imaging. The classic scratch wound assay was used to model wound closure of keratinocytes after inoculation with Bt*.* Bacterial infected cells exhibited a significantly larger wound area at 24 hpi as compared to uninfected and PFA-fixed bacterial treated cells (Figure 1(c)). Live imaging revealed that keratinocytes cultured with Bt closed the scratch wound at a slower rate than uninfected cells (S5 Movie). Our results demonstrate that Bt infection impedes keratinocyte wound closure.

### Infection of keratinocyte monolayer results in cellular extrusion

**Figure 2.**
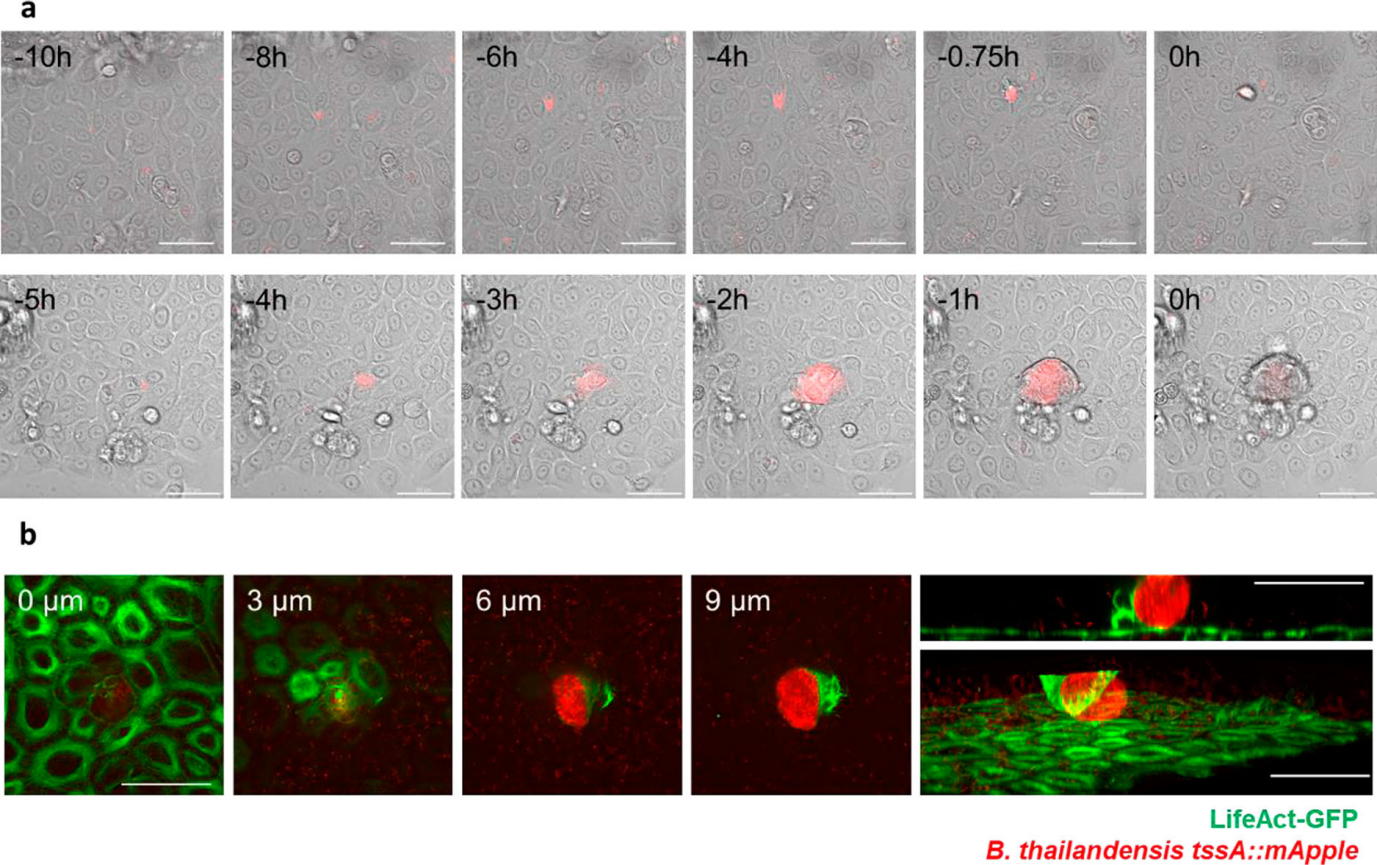
Extrusion of Bt infected keratinocytes. (a). Time-lapse imaging of Bt (in red) infected primary keratinocytes. Extrusion of a single cell (top panel) and a group of cells (bottom panel) are displayed. Timestamp is indicated on the top left corner of each image, with 0 h defined as the time of cell extrusion. Scalebar = 50 µm. (b). *Z*-stack images of extruded Bt (in red) infected N/TERT-1 keratinocytes expressing LifeAct-GFP (in green). Shown in the left corner is the height above the monolayer. Top right most panel shows a cross-section through the infected cell. Bottom right most panel shows 3D-rendering of *Z*-stack. Scale bar = 50 µm.

In the 2D keratinocyte scratch wound assay, we observed that infected cells were being extruded from the keratinocyte monolayer. To examine this further, we employ time-lapse imaging to track keratinocyte infection by Bt*.* We documented the subsequent extrusion and removal of the infected cell from the monolayer (Figure 2(a)). This phenomenon was observed in both single cells (Figure 2(a) top panel, S6 Movie) and clusters of cells (Figure 2(a) bottom panel, S7 Movie). To confirm the extrusion of infected cells, confocal *z*-stack live-imaging was performed*.* An infected cell was clearly visible on the apical side of the keratinocyte monolayer, confirming extrusion (Figure 2(b), S8 Movie).

### *B. thailandensis* infected keratinocytes activate pyroptosis

We wondered whether extruded keratinocytes showed features of pyroptosis, which had been reported in intestinal cells undergoing extrusion [23]. Inflammasome activation is an innate immune pathway involving the assembly of supramolecular complexes in the cytoplasm for proteolytic activation of proinflammatory cytokines driving systemic immune response and inflammation as reviewed [24]. Inflammasomes comprise a sensor protein, inflammatory caspases and in some cases require an adaptor protein such as the apoptosis-associated speck-like protein containing a CARD domain (ASC) [24]. ASC speck formation, a hallmark of inflammasome activation, had been previously observed to precede cell extrusion from the skin epithelia in zebrafish [25].

**Figure 3.**
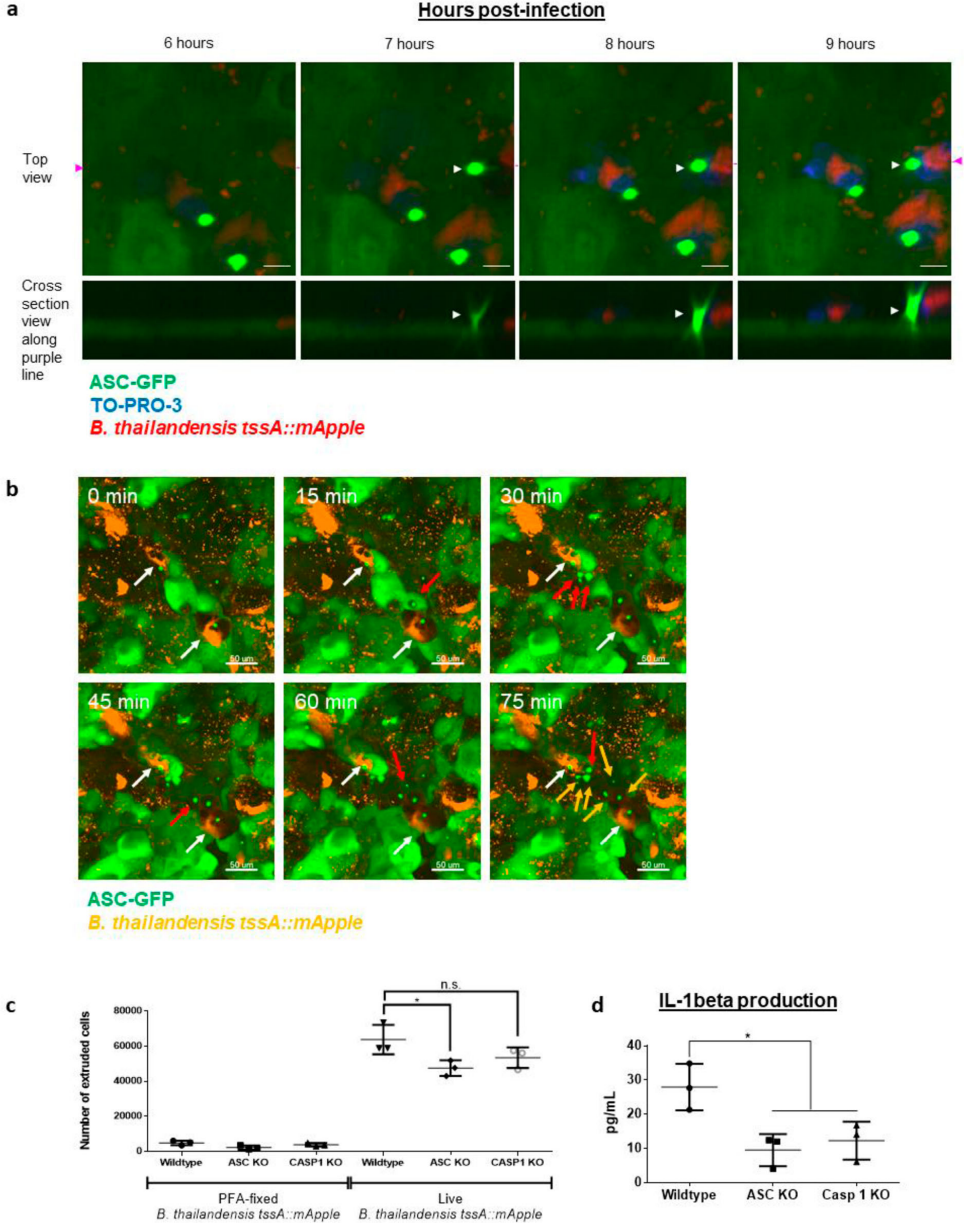
Bt infected keratinocytes activate inflammasomes. (a). Sequence of events showing that Bt infected N/TERT-1 keratinocytes undergo ASC activation, cell death and subsequent cellular extrusion. Bt is displayed in red. Keratinocytes express ASC-GFP, displayed in green. Cell impermeant TO-PRO-3 nucleic acid stain was used to label the nuclei of dead cells and is displayed in blue. White arrows indicate the extruding cell. Scale bar = 10 µm. (b). Time-lapse imaging of Bt infection (orange) of ASC-GFP expressing N/TERT-1 keratinocytes showing ASC activation in cells neighbouring the infected cell. Timescale is indicated on the top left-hand corner of each field-of-view. 0 min is defined as the time when infected cells with ASC speck were observed, prior to ASC activation in neighbouring cells. White arrows indicate infected cells with an ASC speck (orange with green speck). Newly observed bystander cells in the various timepoints are indicated by red arrows. All observed neighbouring cells (non-orange) with ASC speck are indicated with yellow and red arrows in the final 75 min timepoint. Scale bar = 50 µm. (c). Extruded cell counts of Bt infected cells at MOI 200, 48 hpi. PFA-fixed Bt was used as a control. Assay was performed in triplicates and the values expressed as mean ± S.D. (d). IL-1β secretion in Bt infected cells at MOI 200, 24 hpi. Graph presented is a representative of three independent experiments performed, where each datapoint represents the mean value from an independent experiment. Error bars represent SD.

To test for inflammasome activation, we utilized a reporter cell line, N/TERT-1 ASC-GFP expressing keratinocytes, that forms an ASC-GFP positive speck when undergoing canonical inflammasome-driven pyroptosis. We also utilized cell impermeant nucleic acid stain, TO-PRO-3, during live imaging of Bt infected ASC-GFP N/TERT-1 keratinocytes to examine pyroptotic cell death. Bt infected cells underwent ASC speck formation followed by cell death and subsequent cellular extrusion (Figure 3(a), S9 Movie). ASC activation was also observed in cells neighbouring the infected cell (Figure 3(b), S10 Movie), which could be due to low but undetectable levels of infection in the surrounding cells.

We next quantified the extruded cells in wildtype keratinocytes and the corresponding ASC and caspase-1 (CASP1) knockout (KO) cells defective in the pyroptotic signalling pathway. Bt infected ASC KO cells had significantly fewer extruded cells as compared to wildtype cells (Figure 3(c)) indicating that the initiation of pyroptosis via ASC activation leads to cellular extrusion. Bt infected CASP1 KO cells showed lower number of extruded cells but were not statistically different from infected wildtype cells (*p* = 0.1434). However, the counting of extruded cells over time required several experimental steps that could be highly variable and is very low throughput, likely only capturing a small fraction of extrusion events. To document another measure of inflammasome activation, we examined interleukin-1β (IL-1β) secretion. Bt infected ASC and CASP1 KO cells produced significantly lower amounts of IL-1β as compared to wildtype cells, confirming that pyroptosis and its downstream signalling pathways were activated during Bt infection (Figure 3(d)).

### Activation of the inflammasome pathway during *Burkholderia* infection results in reduced bacterial burden

**Figure 4.**
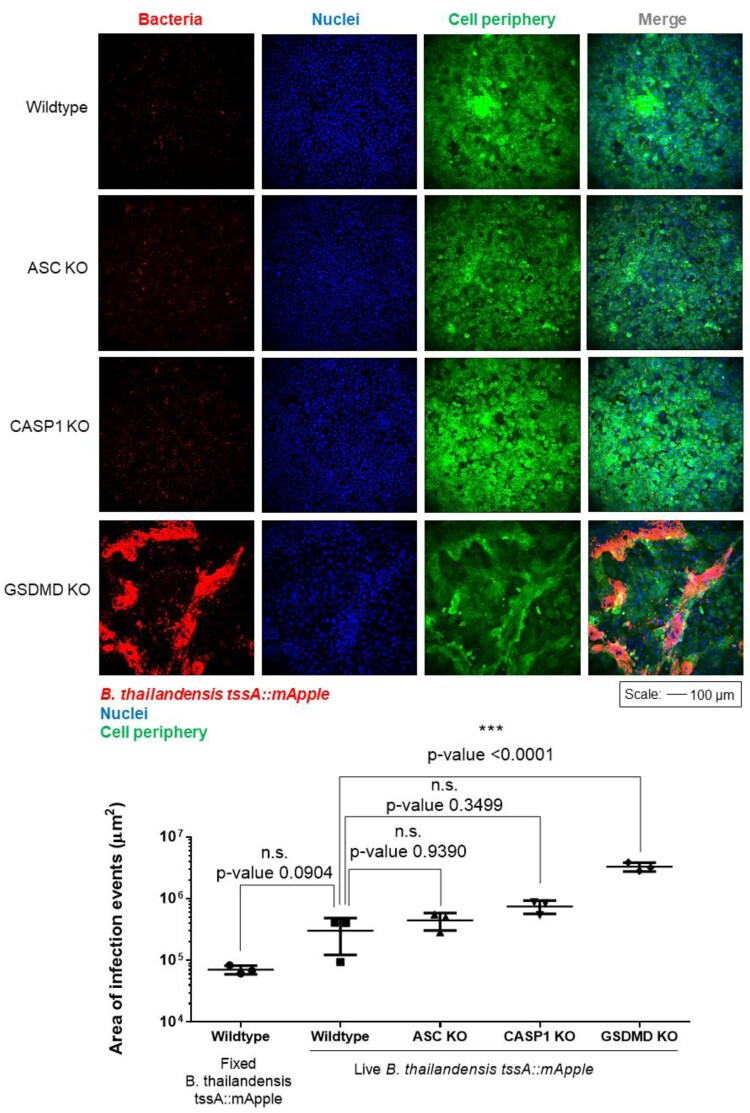
Keratinocytes utilize pyroptosis as a defence mechanism to eliminate infected cells. (a). Bacterial loads of cells infected at MOI 200, 24 hpi. Extracellular bacteria were eliminated via Imaris 3D surface rendering using Cell Mask signals to define intracellular regions. Bt is displayed in red. Cell periphery and nuclei are labelled via Cell Mask and Hoechst staining and are displayed in green and blue respectively. Scale bar = 100 µm. (b). Area of infection was examined from the confocal micrographs based on bacterial fluorescence intensities. Data presented as mean ± S.D from biological triplicates.

We hypothesized that cellular extrusion triggered via inflammasome activation during Bt infection could be a host defense mechanism to eliminate infected cells. To explore this hypothesis, we examined the bacterial burden of Bt-infected pyroptosis-defective cells. We were unable to quantify bacterial loads via serial dilution and plating of intracellular bacteria as the differentiated keratinocytes were resistant to lysis with 1% Triton-X. Therefore, we utilized confocal microscopy to estimate intracellular bacterial loads. Extracellular bacteria were excluded via Imaris 3D surface rendering using Cell Mask staining to delineate extracellular versus intracellular regions. To prevent sampling bias, a large area of 3.57 × 3.57 mm^2^ was acquired via tile scan (Figure S1) and three independent experiments were performed. Bt infected wildtype keratinocytes were observed to have fewer infected cells (Figure 4(a)) and lower total area of infection as compared to infected ASC KO and CASP1 KO cells, although it was not statistically significant (Figure 4(b)). Since gasdermin D (GSDMD) mediates pyroptosis downstream of CASP1 activation, we examined the bacterial burden in infected GSDMD KO cells. Quantification of intracellular bacterial signals also revealed significantly higher number of infection events and larger total area of infection for GSDMD KO cells compared to infected wildtype cells (Figure 4(a,b)).

### Infection of *ex vivo* human skin cultures leads to epidermis detachment

**Figure 5.**
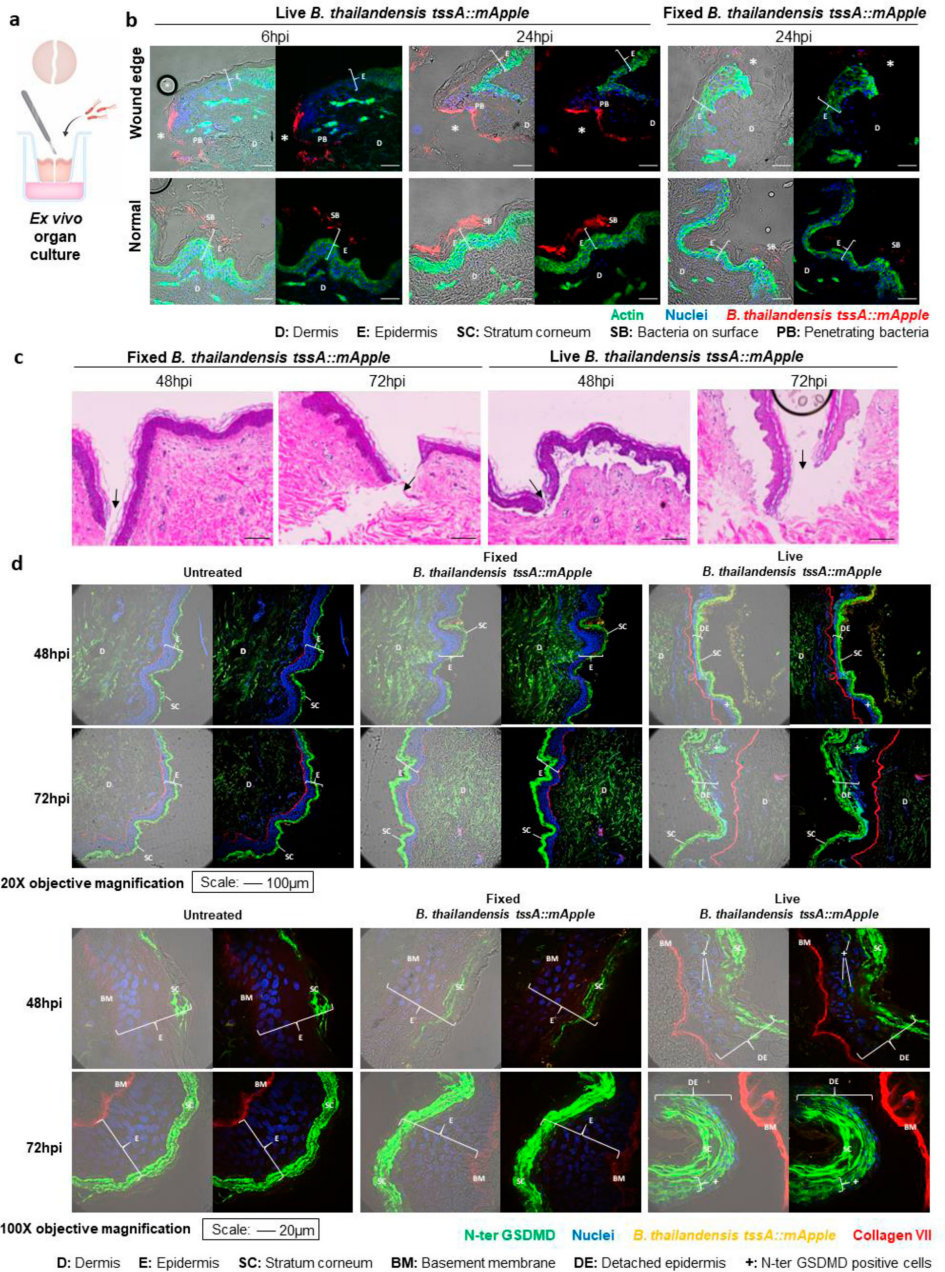
Bt infection of EVOCs exhibits epidermal detachment which corresponds to GSDMD activation. (a). Schematic of *ex vivo* skin culture. (b). EVOCs infected with Bt at MOI 200, 6 and 24 hpi. Cells were permeabilized with Triton-X before staining with phalloidin conjugated AF488 and DAPI. Shown is the cross-section of infected skin cultures with actin in green, Bt in red, and nuclei in blue. DIC is included in the left panel image. PFA-fixed bacteria at 24 hpi were used as a control. Asterisks mark wound edge. Scale bar = 100 µm. D: Dermis, E: Epidermis, SC: Stratum corneum, SB: Surface bacteria, PB: Penetrating bacteria. (c). Cross-sectional H&E images of EVOCs treated with PFA-fixed or infected with Bt at MOI 200 after 48 and 72 hpi. Arrows mark the wound site. Scale bar = 100 µm. (d). EVOCs infected with Bt at MOI200, 48 and 72 hpi. Cryosectioned EVOCs were stained without permeabilization. Shown is a cross-section of skin with N-terminal GSDMD in green, collagen VII in red, bacteria in orange and nuclei in blue. Uninfected EVOCs and PFA-fixed bacteria were included as controls. DIC is included in the left panel image. Images were acquired at 20X and 100X objective magnification and the scale bars correspond to 100 and 20 µm respectively. D: Dermis, E: Epidermis, SC: Stratum corneum, DE: Detached epidermis, BM: Basement membrane, +: N-ter GSDMD positive cells.

Inflammasome activation and cellular extrusion of infected cells in the *in vitro* skin model led us to ask about the possible consequence in patients with a skin infection. We utilized *ex vivo* organ culture (EVOC) of human skin for more relevance to the actual infection circumstances (Figure 5(a)). The skin was wounded by scalpel cut before Bt inoculation and the cross-section of whole skin tissue analyzed. At both 6 and 24 hpi, bacteria were observed to be localized to the wound edge, with increased numbers of bacteria at the wound edge by 24 h (Figure 5(b)). In contrast, EVOCs treated with PFA-fixed Bt had decreased number of bacteria at the wound edge compared to Bt infected cultures at 24 h, confirming that bacteria proliferated at the wound edge. In the undamaged skin sites, bacteria could be seen attaching to the cornified layer of skin with no penetration into the viable epidermal layer beneath (Figure 5(b)), agreeing with our results in Figure 1. At later timepoints (48 and 72 hpi), we observed widespread epidermal detachment from the underlying dermis only in Bt infected cultures and not the controls (Figure 5(c)). Epidermal detachment was evident throughout the entirety of the *ex vivo* skin culture (Figure S2).

To determine if inflammasome activation also occurs in infected EVOCs, we assessed for GSDMD pore formation via immunostaining of Bt infected human skin cultures. Human skin cultures were not permeabilized prior to immunostaining with a commercial GSDMD polyclonal antibody which was generated using the N-terminus of GSDMD (amino acids 1-200) as an antigen. Therefore, the signals obtained from the staining are indicative of cleaved GSDMD which is active and forms pores on the plasma membrane that is accessible to the antibody, and not intracellular full-length GSDMD [24]. Strong signals for cleaved GSDMD were observed in the stratum corneum; the cornified layer of the skin in all our EVOC samples at both 48 and 72 hpi (Figure 5(d), SC). This is likely due to staining of the dead cells in cornified layer. This could mean that the dead cells exhibit cleaved GSDMD on their surface, or that the cornified layer allowed the antibody to enter to cell interior even without permeabilization. Only EVOCs infected with live Bt show positive staining for cleaved GSDMD at 48 hpi and more so at 72 hpi beneath the stratum corneum layer (Figure 5(d)). An isotype control antibody against an intracellular protein, H2AX, did not show staining on the stratum corneum nor beneath the stratum corneum layer of the human skin even in live bacterial infected cells (Figure S3). Cells underlying the cornified layer in EVOCs subjected to PFA-fixed bacteria and uninfected EVOCs also did not display GSDMD activation (Figure 5(d)). This indicates that the positive signals with anti-GSDMD antibody beneath the cornified layer represent plasma membrane localized GSDMD (Figure 5(d)) and is not due to non-specific staining. These results demonstrate that Bt infection of the human skin leads to inflammasome activation.

## Discussion

**Figure 6.**
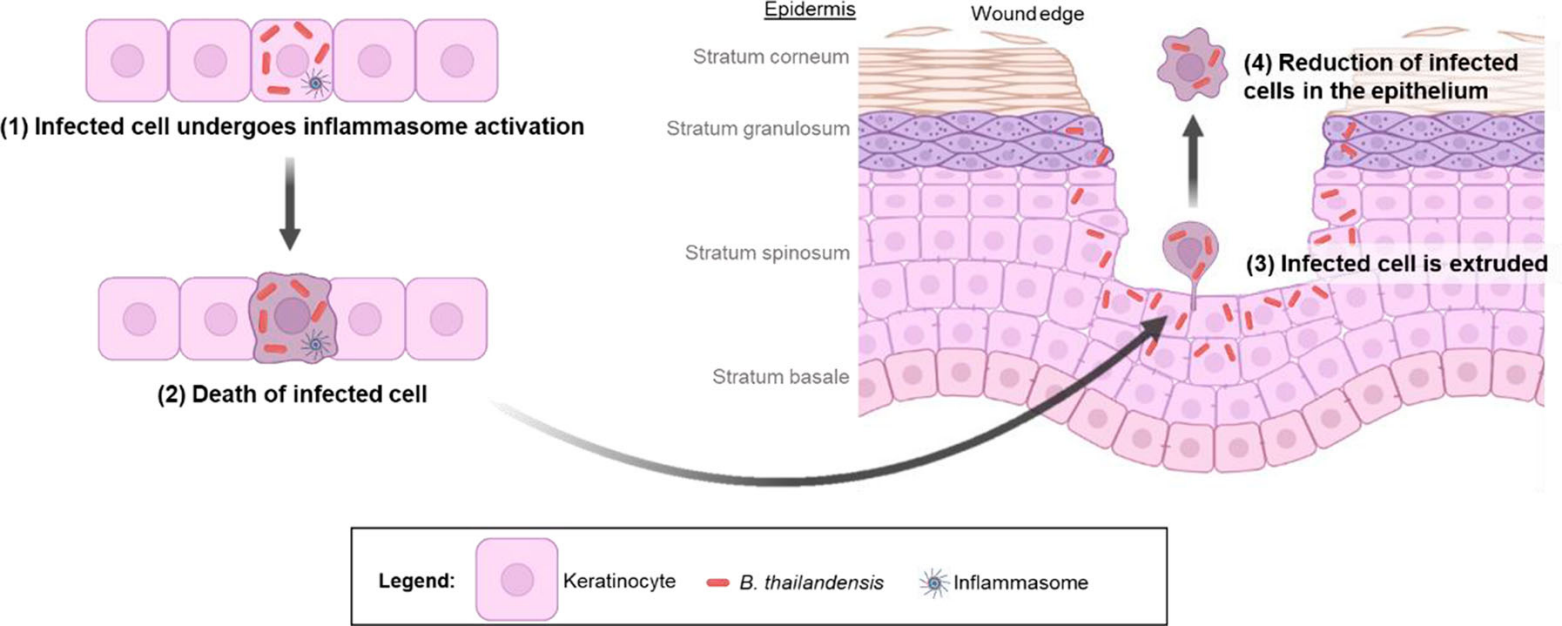
Schematic of the proposed strategy for elimination of *Burkholderia* infection at wound site. This figure was created with BioRender.

Cell extrusion is a process employed by the body to remove dying, dead or unwanted cells from an epithelium while maintaining continuity in the function of the tissue [26,27]. Epithelial tissues cover external surfaces of the body and line internal surfaces, serving as a barrier and the first line of defense against invading pathogens. Cell extrusion has been regarded as a defense mechanism to eliminate infected cells and prevent bacterial invasion [26]. For example, inflammasome components NAIPs 1-6 and NLRC4, caspase-1 and -4 were shown to drive the extrusion of *Salmonella* Typhimurium infected enterocytes into the lumen and limit intraepithelial bacterial loads [23]. Non-canonical inflammasome caspase-4 limited *S.* Typhimurium loads via cell extrusion [28]. Interestingly, our 2D keratinocyte findings parallel the results from these studies. The slower rates of gap closure during our scratch wound assay with live Bt compared to uninfected cells may also be contributed by cellular extrusion since the pool of cells from which keratinocytes proliferate from is also depleted. However, cells would be extruded into the lumen in the intestinal epithelium whereas in the skin, extrusion of the keratinocytes in the stratum spinosum at the wound edge would likely be into the space created by the wound (Figure 6). In the skin, the epidermis is formed by differentiating keratinocytes undergoing cornification, which is a type of cell death, leading to dead corneocytes forming the skin’s barrier. It thus makes sense that infection could only proceed via a break in the skin epidermis, which is otherwise a stratified epithelium with a cornified outer layer, followed by keratinocytes in the stratum granulosum and stratum spinosum. Our 3D skin equivalent and *ex vivo* skin models revealed that *Burkholderia* infection does not progress into the deeper layers of the epidermis unless there is a wound. This demonstrates that the cornified layer serves as an effective barrier preventing bacterial invasion. Prolonged *Burkholderia* infection of the *ex vivo* skin cultures, however, led to epidermal detachment, which could be reflective of skin ulceration, referring to a circumscribed loss of the epidermis and portions of the dermis. This is one of the most common presentations of cutaneous melioidosis [11,29–31]. It is unclear what led to epidermal detachment. We postulate that GSDMD activation in the epidermal layers beneath the stratum corneum during prolonged *Burkholderia* infection could trigger extensive cell death and extrusion of the keratinocytes into the stratum corneum. This may create an upward push leading to a loss of integrity and detachment. Of note, GSDMD gene expression is downregulated during keratinocyte differentiation as keratinocytes move upwards from stratum basale to the stratum corneum [32]. It is possible that the cornified layer acts as a physical barrier to prevent bacterial invasion while the underlying epidermal layers utilize GSDMD as a protective mechanism to eliminate bacterial infected cells. Bacterial infection persisting beneath the stratum corneum could lead to extensive GSDMD activity, potentially leading to a loss of the epidermis.

We have shown that cellular extrusion of infected keratinocytes is preceded by inflammasome activation. The expulsion of cells during *Burkholderia* infection is dependent, at least partially, on the canonical inflammasome pathway as infected ASC and CASP1 KO cells had approximately 1/3 fewer extruded cells compared to infected wildtype cells (Figure 3(c)). The partial reduction indicates that other cell death pathways contribute to cellular extrusion during *Burkholderia* infection. However, it could also reflect the difficulty in quantifying the number of extruded cells through our assay due to loss of cells during preparation. In fact, although extrusion of infected cells was documented consistently, it was difficult to quantify accurately due to significant sample variation. This makes it difficult to determine contribution of various inflammasome pathways to the process, which is the limitation of this study. Bp and Bt can activate the NLRP3 inflammasome [33,34]. *Burkholderia* type III secretion rod (BsaL) and needle (BsaK) proteins were also shown to trigger the activation of NLRC4 [35–39]. Studies have delineated the roles of caspases-1 and -4 to reveal their protective functions against *Burkholderia* infection [34,40]. Activation of GSDMD and inflammasome dependent pyroptosis is protective against *Burkholderia* infection in mice, with GSDMD also functioning to directly kill *Burkholderia* [33,41]. Furthermore, caspase-4 triggered pyroptosis in neutrophils [42] and in lung epithelial cells [43] are crucial in defending against Bt. Our observation of the ASC and CASP1 independent activation of cellular extrusion in the 2D keratinocyte model may be a result of caspase-4 triggered pyroptosis, since human keratinocytes had been reported to express caspase-4 [44,45]. Furthermore, interferon gamma-primed keratinocyte cells HaCaT restricted Bt induced multinucleated giant cell formation via guanylate binding protein-1 dependent activation of caspase-4 [46]. The NLRP1 inflammasome is the major inflammasome expressed by keratinocytes *in vitro* and *in vivo* in the human skin under normal and homeostatic conditions [47]. However, under various conditions of stress, NLRP3 and NLRC4 could also be expressed [48–51]. It is possible that *Burkholderia* infection triggers stress that activates NLRP1 inflammasome activation, leading to ASC and CASP1 activation, although the participation of other inflammasomes such as the non-canonical caspase-4 as mentioned previously could not be ruled out.

Notwithstanding the technical difficulties with quantifying extruded cells and the precise role of each inflammasome, we focused on the consequence of failing to activate the inflammasomes by using GSDMD KO cells, GSDMD being the common downstream step in the cell death pathway. *Burkholderia* infected GSDMD KO cells showed the highest and most significant difference in bacterial burden compared to wildtype-infected, ASC or CASP1 KO infected cells, indicating that GSDMD is the main mediator for reducing bacterial loads during *Burkholderia* infection in our human keratinocyte model. Lower bacterial burdens in infected wildtype cells compared to inflammasome defective cells suggests that inflammasome activation promotes cellular extrusion and cell death, thereby restricting *Burkholderia* replication in the monolayer. Although the overall bacterial burden within the epithelial tissue could be reduced through cellular extrusion, it remains to be determined whether the expulsion of *Burkholderia* infected cells is beneficial to the host. The 3D organotypic and EVOC models used in this study are largely able to recapitulate the skin epidermis but not the function of the dermis; which includes structures such as the sebaceous glands and vasculature. Certain lipids present in sebum secreted from sebaceous glands function to maintain skin barrier integrity and has been previously reported to exhibit antimicrobial activity [52]. It is also possible that the extruded cells are eliminated by the immune cells infiltrating into the wound site, limiting bacterial propagation. Further investigations using *in vivo* skin infection models are required to track the fate of extruded infected keratinocytes in the context of a wound.

Another potential limitation of our models is the use of Bt instead of Bp. Although Bt is considered relatively avirulent, there are documentations of infected patients manifesting symptoms of melioidosis with several reports of Bt causing skin infections [53–56]. Bt E264 isolate chosen for this study is the most common strain utilized for investigating molecular mechanisms of Bt infection and is virulent in several animal models although higher infectious doses are required as compared to Bp [57,58]. Previous phylogenetic analyses of Bt strains revealed a distinct phylogenetic clade consisting of pleural wound isolated CDC2721121, forearm wound isolated BtAR2017 and blood isolated TxDOH strain – as well as capsular polysaccharide-producing strain E555 [54,59]. Although it was postulated that members of this cluster could be more virulent than other Bt strains, both Bt E264 and E555 were virulent in BALB/c mice via intraperitoneal infection [59]. In a Syrian hamster model, E264 and TxDOH were virulent, killing all the hamsters within one week of challenge at various doses, whereas CDC2721121 was avirulent in this model [57]. E264 was also reported to be more virulent in BALB/c in an intraperitoneal challenge compared to TxDOH and CDC2721121 [57]. Thus, Bt E264 can be considered a good surrogate for Bp infection especially when infection is conducted with a high dose as we have done.

In summary, our work presents a novel aspect of *Burkholderia* pathogenesis at the skin and highlights the importance of using appropriate systems to better model different disease presentations. 3D cultures and EVOCs represent more physiological and realistic systems to study host–pathogen interactions at the skin interface and could yield important insights that might otherwise be overlooked.

## Supplementary Material

Supplemental Material

## References

[1] Wiersinga WJ, Virk HS, Torres AG, et al. Melioidosis. Nat Rev Dis Primers. 2018 Feb 1;4:17107. 29388572 10.1038/nrdp.2017.107 PMC6456913

[2] Cheng AC, Currie BJ. Melioidosis: epidemiology, pathophysiology, and management. Clin Microbiol Rev. 2005 Apr;18(2):383–416. 15831829 10.1128/CMR.18.2.383-416.2005 PMC1082802

[3] Limmathurotsakul D, Wongsuvan G, Aanensen D, et al. Melioidosis caused by *Burkholderia pseudomallei* in drinking water, Thailand, 2012. Emerg Infect Dis. 2014 Feb;20(2):265–268. 24447771 10.3201/eid2002.121891 PMC3901481

[4] Kaestli M, Schmid M, Mayo M, et al. Out of the ground: aerial and exotic habitats of the melioidosis bacterium Burkholderia pseudomallei in grasses in Australia. Environ Microbiol. 2012 Aug;14(8):2058–2070. 22176696 10.1111/j.1462-2920.2011.02671.x PMC3319007

[5] Limmathurotsakul D, Kanoksil M, Wuthiekanun V, et al. Activities of daily living associated with acquisition of melioidosis in northeast Thailand: a matched case-control study. PLoS Negl Trop Dis. 2013;7(2):e2072. 23437412 10.1371/journal.pntd.0002072 PMC3578767

[6] Currie BJ, Ward L, Cheng AC. The epidemiology and clinical spectrum of melioidosis: 540 cases from the 20 year Darwin prospective study. PLoS Negl Trop Dis. 2010;4(11):e900. 21152057 10.1371/journal.pntd.0000900 PMC2994918

[7] Suputtamongkol Y, Hall AJ, Dance DA, et al. The epidemiology of melioidosis in Ubon Ratchatani, northeast Thailand. Int J Epidemiol. 1994 Oct;23(5):1082–1090. 7860160 10.1093/ije/23.5.1082

[8] Suputtamongkol Y, Chaowagul W, Chetchotisakd P, et al. Risk factors for melioidosis and bacteremic melioidosis. Clin Infect Dis. 1999 Aug;29(2):408–413. 10476750 10.1086/520223

[9] Currie BJ, Fisher DA, Howard DM, et al. The epidemiology of melioidosis in Australia and Papua New Guinea. Acta Trop. 2000 Feb 5;74(2-3):121–127. 10674639 10.1016/s0001-706x(99)00060-1

[10] Fertitta L, Vignon-Pennamen MD, Frazier A, et al. Necrobiosis lipoidica with bone involvement successfully treated with infliximab. Rheumatology. 2019 Sep 1;58(9):1702–1703. 30877774 10.1093/rheumatology/kez055

[11] Gibney KB, Cheng AC, Currie BJ. Cutaneous melioidosis in the tropical top end of Australia: a prospective study and review of the literature. Clin Infect Dis. 2008 Sep 1;47(5):603–609. 18643756 10.1086/590931

[12] Soffler C, Bosco-Lauth AM, Aboellail TA, et al. Pathogenesis of percutaneous infection of goats with *Burkholderia pseudomallei*: clinical, pathologic, and immunological responses in chronic melioidosis. Int J Exp Pathol. 2014 Apr;95(2):101–119. 24571408 10.1111/iep.12068 PMC3960038

[13] Whiteley L, Meffert T, Haug M, et al. Entry, intracellular survival, and multinucleated-giant-cell-forming activity of *Burkholderia pseudomallei* in human primary phagocytic and nonphagocytic cells. Infect Immun. 2017 Oct;85:10. 10.1128/IAI.00468-17 PMC5607410 28760929

[14] Brett PJ, DeShazer D, Woods DE. *Burkholderia thailandensis* sp. nov., a *Burkholderia pseudomallei*-like species. Int J Syst Bacteriol. 1998 Jan;48(1):317–320. 9542103 10.1099/00207713-48-1-317

[15] French CT, Toesca IJ, Wu TH, et al. Dissection of the Burkholderia intracellular life cycle using a photothermal nanoblade. Proc Natl Acad Sci U S A. 2011 Jul 19;108(29):12095–12100. 21730143 10.1073/pnas.1107183108 PMC3141958

[16] Schell MA, Ulrich RL, Ribot WJ, et al. Type VI secretion is a major virulence determinant in *Burkholderia mallei*. Mol Microbiol. 2007 Jun;64(6):1466–1485. 17555434 10.1111/j.1365-2958.2007.05734.x

[17] Kim HS, Schell MA, Yu Y, et al. Bacterial genome adaptation to niches: divergence of the potential virulence genes in three Burkholderia species of different survival strategies. BMC Genom. 2005 Dec 7;6:174. 10.1186/1471-2164-6-174 PMC1343551 16336651

[18] Haraga A, West TE, Brittnacher MJ, et al. *Burkholderia thailandensis* as a model system for the study of the virulence-associated type III secretion system of *Burkholderia pseudomallei*. Infect Immun. 2008 Nov;76(11):5402–5411. 18779342 10.1128/IAI.00626-08 PMC2573339

[19] Jimenez V J, Moreno R, Kaufman E, et al. Effects of binge alcohol exposure on *Burkholderia thailandensis*-alveolar macrophage interaction. Alcohol. 2017 Nov;64:55–63. 28965656 10.1016/j.alcohol.2017.04.004

[20] Whiteley L, Haug M, Klein K, et al. Cholesterol and host cell surface proteins contribute to cell-cell fusion induced by the Burkholderia type VI secretion system 5. PLoS One. 2017;12(10):e0185715. 28973030 10.1371/journal.pone.0185715 PMC5626464

[21] Ku JWK, Chen Y, Lim BJW, et al. Bacterial-induced cell fusion is a danger signal triggering cGAS-STING pathway via micronuclei formation. Proc Natl Acad Sci U S A. 2020 Jul 7;117(27):15923–15934. 32571920 10.1073/pnas.2006908117 PMC7355030

[22] Sokolov I, Guz NV, Iyer S, et al. Recovery of aging-related size increase of skin epithelial cells: in vivo mouse and in vitro human study. PLoS One. 2015;10(3):e0122774. 25807526 10.1371/journal.pone.0122774 PMC4373822

[23] Sellin ME, Muller AA, Felmy B, et al. Epithelium-intrinsic NAIP/NLRC4 inflammasome drives infected enterocyte expulsion to restrict Salmonella replication in the intestinal mucosa. Cell Host Microbe. 2014 Aug 13;16(2):237–248. 25121751 10.1016/j.chom.2014.07.001

[24] Zheng D, Liwinski T, Elinav E. Inflammasome activation and regulation: toward a better understanding of complex mechanisms. Cell Discov. 2020;6:36. 32550001 10.1038/s41421-020-0167-x PMC7280307

[25] Kuri P, Schieber NL, Thumberger T, et al. Dynamics of in vivo ASC speck formation. J Cell Biol. 2017 Sep 4;216(9):2891–2909. 28701426 10.1083/jcb.201703103 PMC5584180

[26] Gudipaty SA, Rosenblatt J. Epithelial cell extrusion: pathways and pathologies. Semin Cell Dev Biol. 2017 Jul;67:132–140. 27212253 10.1016/j.semcdb.2016.05.010 PMC5116298

[27] Rosenblatt J, Raff MC, Cramer LP. An epithelial cell destined for apoptosis signals its neighbors to extrude it by an actin- and myosin-dependent mechanism. Curr Biol. 2001 Nov 27;11(23):1847–1857. 11728307 10.1016/s0960-9822(01)00587-5

[28] Knodler LA, Crowley SM, Sham HP, et al. Noncanonical inflammasome activation of caspase-4/caspase-11 mediates epithelial defenses against enteric bacterial pathogens. Cell Host Microbe. 2014 Aug 13;16(2):249–256. 25121752 10.1016/j.chom.2014.07.002 PMC4157630

[29] Currie BJ, Fisher DA, Howard DM, et al. Endemic melioidosis in tropical northern Australia: a 10-year prospective study and review of the literature. Clin Infect Dis. 2000 Oct;31(4):981–986. 11049780 10.1086/318116

[30] Fertitta L, Monsel G, Torresi J, et al. Cutaneous melioidosis: a review of the literature. Int J Dermatol. 2019 Feb;58(2):221–227. 30132827 10.1111/ijd.14167

[31] Linton CP. Essential morphologic terms and definitions. J Dermatol Nurses Assoc. 2011;3(2):102–103.

[32] Lachner J, Mlitz V, Tschachler E, et al. Epidermal cornification is preceded by the expression of a keratinocyte-specific set of pyroptosis-related genes. Sci Rep. 2017 Dec 12;7(1):17446. 29234126 10.1038/s41598-017-17782-4 PMC5727156

[33] Ceballos-Olvera I, Sahoo M, Miller MA, et al. Inflammasome-dependent pyroptosis and IL-18 protect against *Burkholderia pseudomallei* lung infection while IL-1beta is deleterious. PLoS Pathog. 2011 Dec;7(12):e1002452. 22241982 10.1371/journal.ppat.1002452 PMC3248555

[34] Aachoui Y, Kajiwara Y, Leaf IA, et al. Canonical inflammasomes drive IFN-gamma to prime caspase-11 in defense against a cytosol-invasive bacterium. Cell Host Microbe. 2015 Sep 9;18(3):320–332. 26320999 10.1016/j.chom.2015.07.016 PMC4567510

[35] Miao EA, Mao DP, Yudkovsky N, et al. Innate immune detection of the type III secretion apparatus through the NLRC4 inflammasome. Proc Natl Acad Sci U S A. 2010 Feb 16;107(7):3076–3080. 20133635 10.1073/pnas.0913087107 PMC2840275

[36] Yang J, Zhao Y, Shi J, et al. Human NAIP and mouse NAIP1 recognize bacterial type III secretion needle protein for inflammasome activation. Proc Natl Acad Sci U S A. 2013 Aug 27;110(35):14408–14413. 23940371 10.1073/pnas.1306376110 PMC3761597

[37] Sun GW, Lu J, Pervaiz S, et al. Caspase-1 dependent macrophage death induced by Burkholderia pseudomallei. Cell Microbiol. 2005 Oct;7(10):1447–1458. 16153244 10.1111/j.1462-5822.2005.00569.x

[38] Bast A, Krause K, Schmidt IH, et al. Caspase-1-dependent and -independent cell death pathways in *Burkholderia pseudomallei* infection of macrophages. PLoS Pathog. 2014 Mar;10(3):e1003986. 24626296 10.1371/journal.ppat.1003986 PMC3953413

[39] Lichtenegger S, Stiehler J, Saiger S, et al. *Burkholderia pseudomallei* triggers canonical inflammasome activation in a human primary macrophage-based infection model. PLoS Negl Trop Dis. 2020 Nov;14(11):e0008840. 33137811 10.1371/journal.pntd.0008840 PMC7605897

[40] Aachoui Y, Leaf IA, Hagar JA, et al. Caspase-11 protects against bacteria that escape the vacuole. Science. 2013 Feb 22;339(6122):975–978. 23348507 10.1126/science.1230751 PMC3697099

[41] Wang J, Deobald K, Re F. Gasdermin D protects from melioidosis through pyroptosis and direct killing of bacteria. J Immunol. 2019 Jun 15;202(12):3468–3473. 31036765 10.4049/jimmunol.1900045 PMC6548608

[42] Kovacs SB, Oh C, Maltez VI, et al. Neutrophil caspase-11 is essential to defend against a cytosol-invasive bacterium. Cell Rep. 2020 Jul 28;32(4):107967. 32726630 10.1016/j.celrep.2020.107967 PMC7480168

[43] Wang J, Sahoo M, Lantier L, et al. Caspase-11-dependent pyroptosis of lung epithelial cells protects from melioidosis while caspase-1 mediates macrophage pyroptosis and production of IL-18. PLoS Pathog. 2018 May;14(5):e1007105. 29791511 10.1371/journal.ppat.1007105 PMC5988316

[44] Sollberger G, Strittmatter GE, Kistowska M, et al. Caspase-4 is required for activation of inflammasomes. J Immunol. 2012 Feb 15;188(4):1992–2000. 22246630 10.4049/jimmunol.1101620

[45] Grimstad O, Husebye H, Espevik T. TLR3 mediates release of IL-1beta and cell death in keratinocytes in a caspase-4 dependent manner. J Dermatol Sci. 2013 Oct;72(1):45–53. 23845419 10.1016/j.jdermsci.2013.05.006

[46] Dilucca M, Ramos S, Shkarina K, et al. Guanylate-binding protein-dependent noncanonical inflammasome activation prevents *Burkholderia thailandensis*-induced multinucleated giant cell formation. mBio. 2021 Aug 31;12(4):e0205421. 34399626 10.1128/mBio.02054-21 PMC8406320

[47] Zhong FL, Mamai O, Sborgi L, et al. Germline NLRP1 mutations cause skin inflammatory and cancer susceptibility syndromes via inflammasome activation. Cell. 2016 Sep 22;167(1):187–202. 27662089 10.1016/j.cell.2016.09.001

[48] Zhang C, Xiao C, Dang E, et al. CD100-Plexin-B2 promotes the inflammation in psoriasis by activating NF-kappaB and the inflammasome in keratinocytes. J Invest Dermatol. 2018 Feb;138(2):375–383. 28927892 10.1016/j.jid.2017.09.005

[49] Dai X, Tohyama M, Murakami M, et al. Epidermal keratinocytes sense dsRNA via the NLRP3 inflammasome, mediating interleukin (IL)-1beta and IL-18 release. Exp Dermatol. 2017 Oct;26(10):904–911. 28266737 10.1111/exd.13334

[50] Hung SJ, Tang SC, Liao PY, et al. Photoprotective potential of glycolic acid by reducing NLRC4 and AIM2 inflammasome complex proteins in UVB radiation-induced normal human epidermal keratinocytes and mice. DNA Cell Biol. 2017 Feb;36(2):177–187. 28112987 10.1089/dna.2016.3471

[51] Hiruma J, Harada K, Motoyama A, et al. Key component of inflammasome, NLRC4, was identified in the lesional epidermis of psoriatic patients. J Dermatol. 2018 Aug;45(8):971–977. 29797527 10.1111/1346-8138.14478

[52] Miller SJ, Aly R, Shinefeld HR, et al. In vitro and in vivo antistaphylococcal activity of human stratum corneum lipids. Arch Dermatol. 1988 Feb;124(2):209–215. 3341800

[53] Zueter AM, Abumarzouq M, Yusof MI, et al. Osteoarticular and soft-tissue melioidosis in Malaysia: clinical characteristics and molecular typing of the causative agent. J Infect Dev Ctries. 2017 Jan 30;11(1):28–33. 28141587 10.3855/jidc.7612

[54] Gee JE, Elrod MG, Gulvik CA, et al. *Burkholderia thailandensis* isolated from infected wound, Arkansas, USA. Emerg Infect Dis. 2018 Nov;24(11):2091–2094. 30334705 10.3201/eid2411.180821 PMC6199988

[55] Zueter AR, Abumarzouq M, Yean CY, et al. Skin infection caused by *Burkholderia thailandensis*: case report with review. J Microbiol Infect Dis. 2016;6(2):92–95.

[56] Stavrou C, Veraitch O, Morris-Jones S, et al. Leg ulceration due to cutaneous melioidosis in a returning traveller. BMJ Case Rep. 2021 Jun 14;14:6. 10.1136/bcr-2020-241490 PMC8204165 34127500

[57] Deshazer D. Virulence of clinical and environmental isolates of *Burkholderia oklahomensis* and *Burkholderia thailandensis* in hamsters and mice. FEMS Microbiol Lett. 2007 Dec;277(1):64–69. 17986086 10.1111/j.1574-6968.2007.00946.x

[58] Brett PJ, Deshazer D, Woods DE. Characterization of *Burkholderia pseudomallei* and *Burkholderia pseudomallei*-like strains. Epidemiol Infect. 1997 Apr;118(2):137–148. 9129590 10.1017/s095026889600739x PMC2808781

[59] Sim BM, Chantratita N, Ooi WF, et al. Genomic acquisition of a capsular polysaccharide virulence cluster by non-pathogenic Burkholderia isolates. Genome Biol. 2010;11(8):R89. 20799932 10.1186/gb-2010-11-8-r89 PMC2945791

