# Supplementary material for: Skin models for cutaneous melioidosis reveal Burkholderia infection dynamics at wound’s edge with inflammasome activation, keratinocyte extrusion and epidermal detachment
Source: Emerg Microbes Infect. 2021 Dec 6;10(1):2326–39. doi: 10.1080/22221751.2021.2011621 (PMC8654412; doi:10.1080/22221751.2021.2011621)
Supplement: Supplemental Material [file TEMI_A_2011621_SM3880.docx]

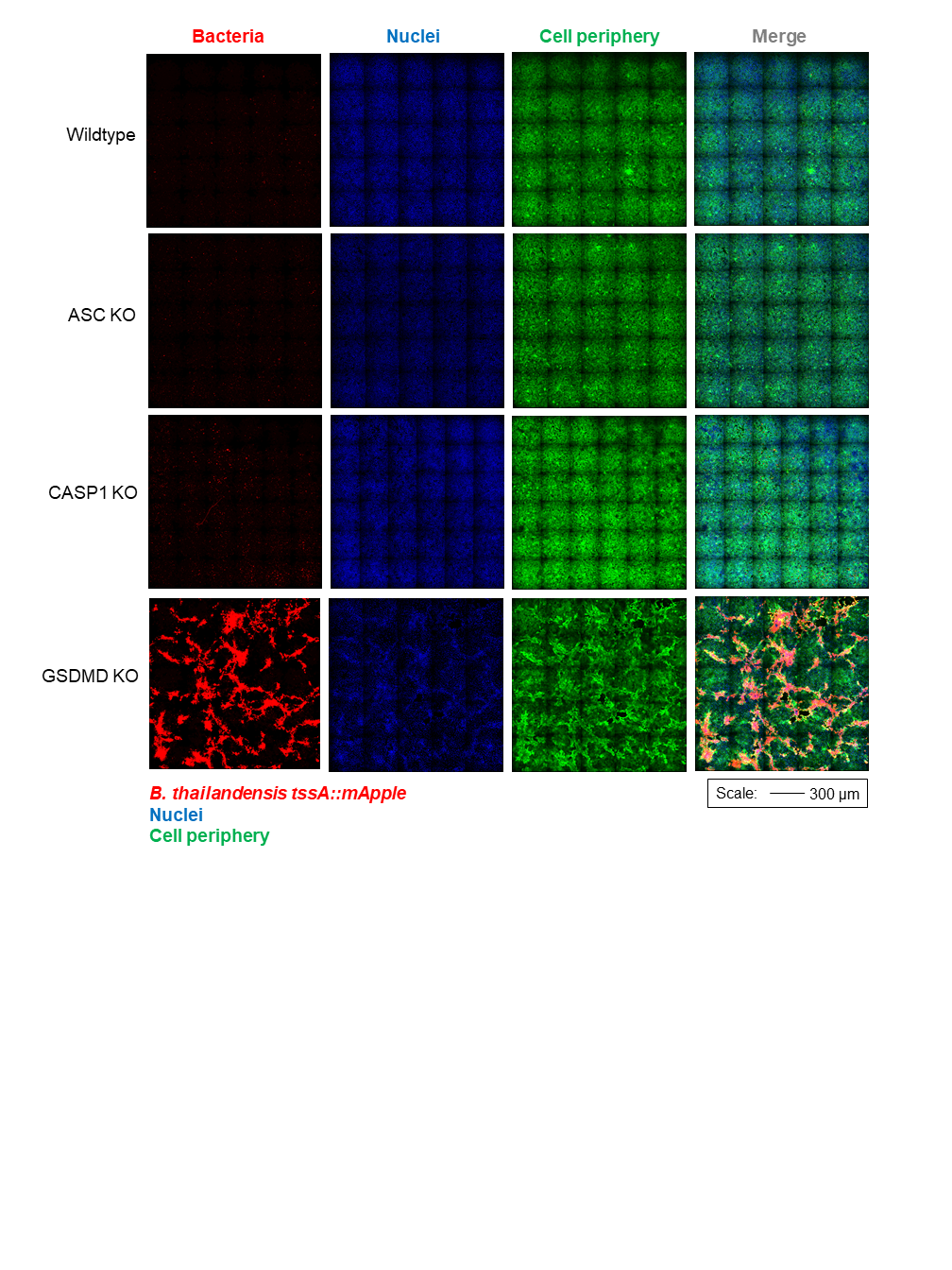


**Supp Fig 1.** Higher bacterial loads were observed in confocal micrographs of Bt infected GSDMD KO keratinocytes as compared to wildtype cells. Cells were infected at MOI 200 and examined for bacterial loads at 24hpi. A large area of 3.57 mm x 3.57 mm was acquired via tile scan and three independent experiments were performed. Extracellular bacteria were eliminated via Imaris 3D surface rendering using Cell Mask signals to define intracellular regions. Bt express mApple chromosomally and are displayed in red. The cell periphery and nuclei were labelled via CellMask and Hoechst staining and are displayed in green and blue respectively. Scalebar = 300µm.


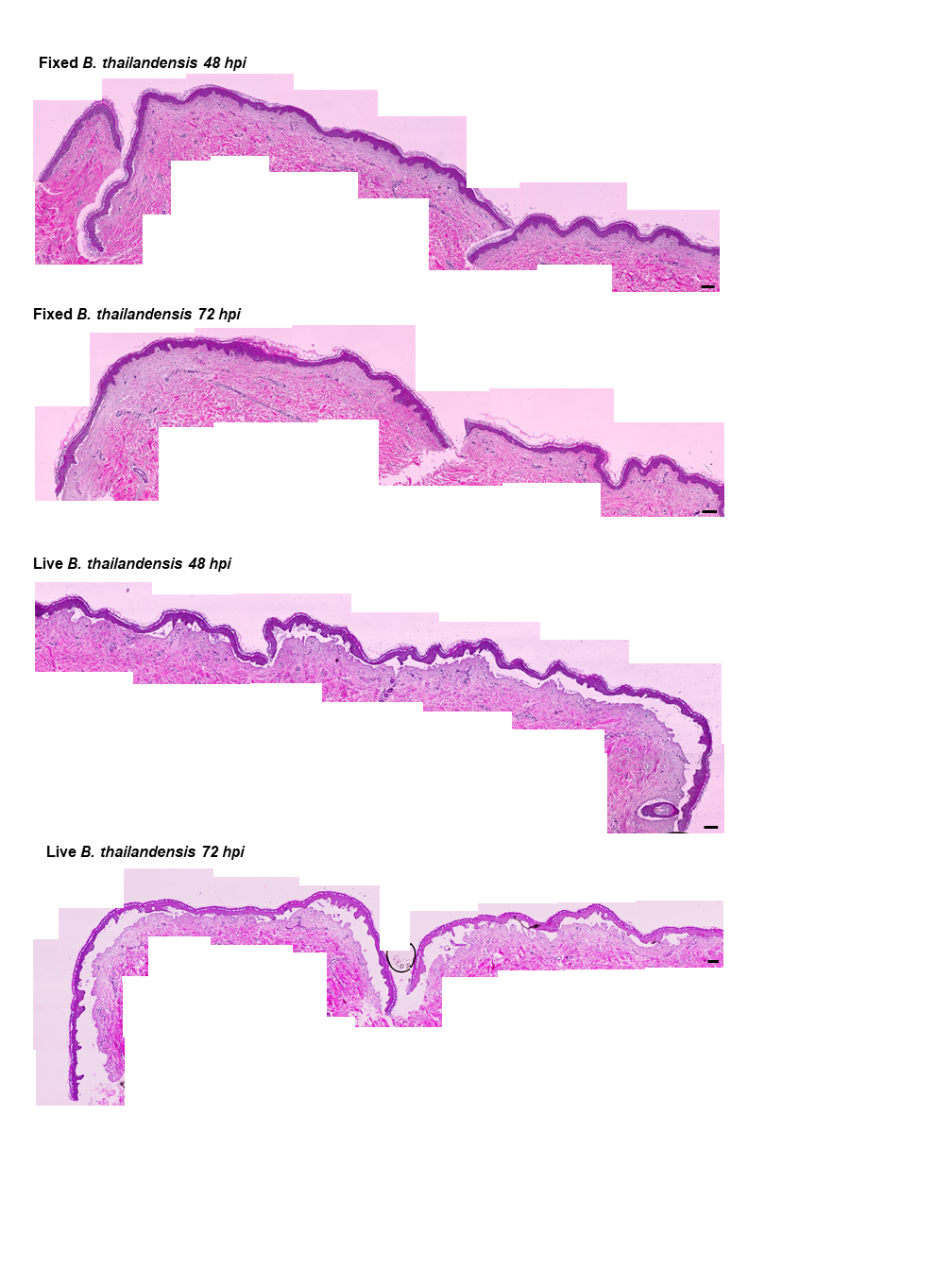


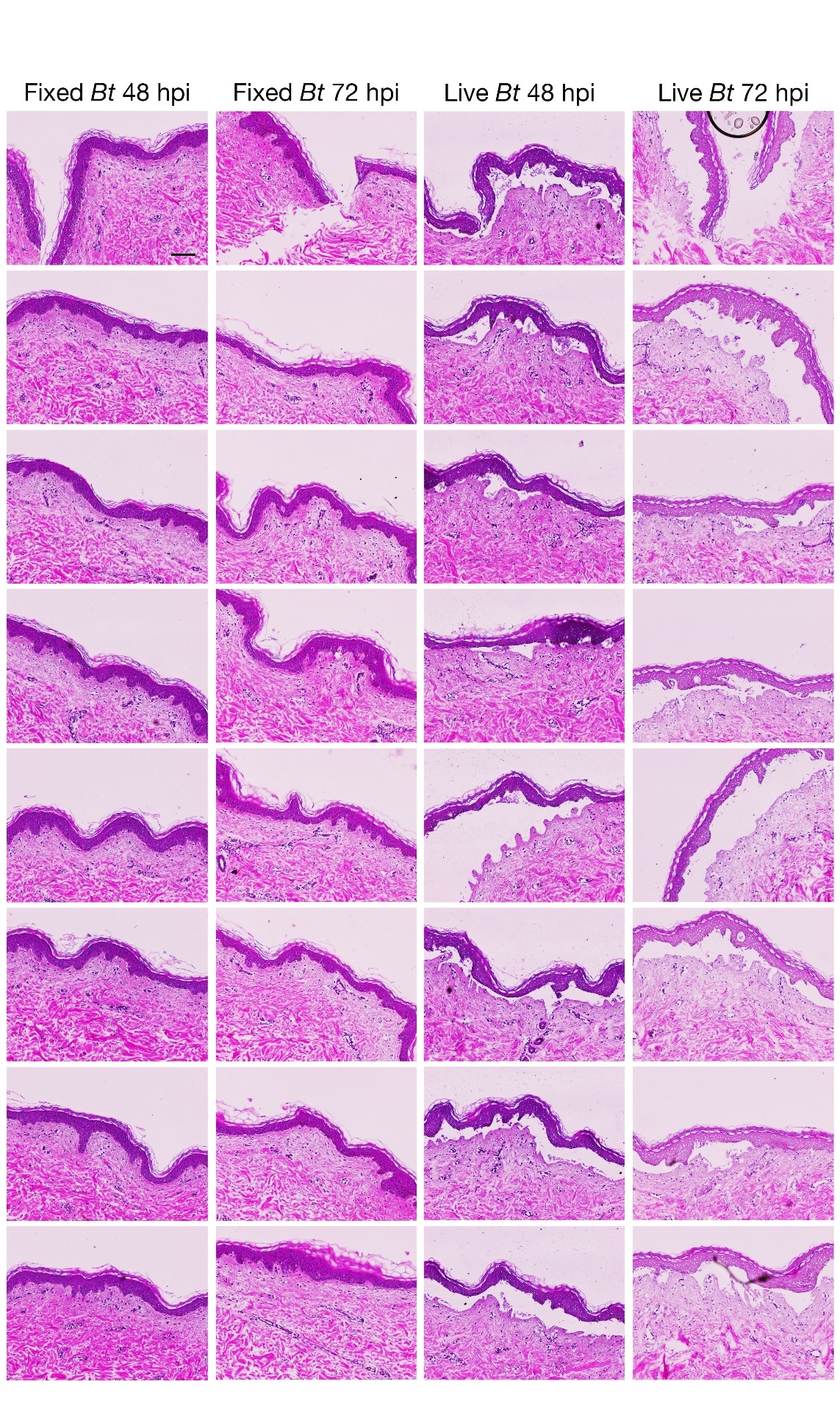


**Supp Fig 2.** Epidermal detachment in *ex vivo* skin culture. Cross-sectional H&E images of *ex vivo* human skin cultures with either PFA-fixed or live mApple labelled Bt after 48 and 72hpi. Scalebar = 100µm.

**
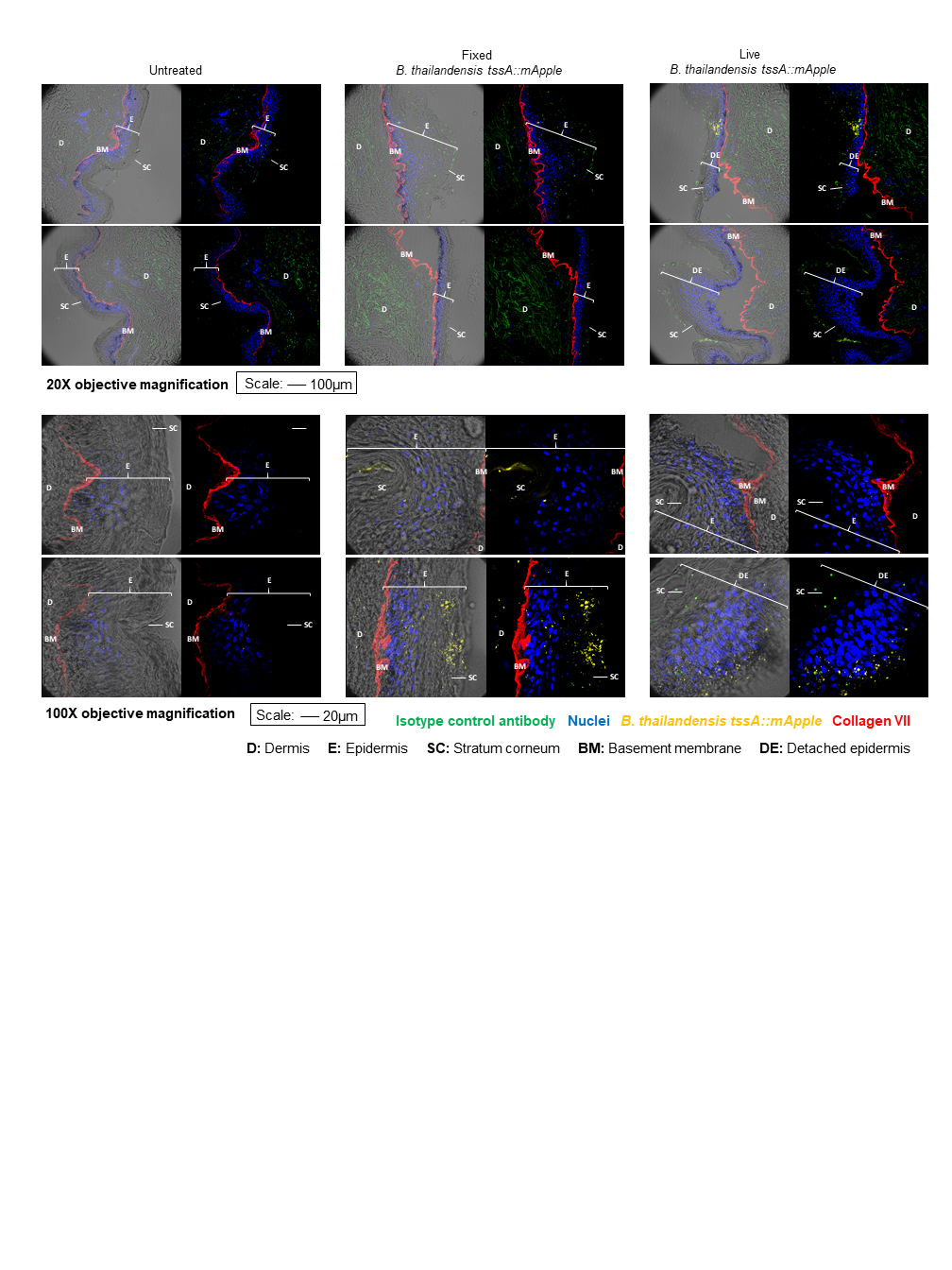
**

**Supp Fig 3.** *Ex vivo* human skin culture infected with mApple labelled Bt stained with isotype control antibodies. Cultures were frozen and fixed at 72hpi. Cryosectioned EVOCs were stained without permeabilization, as in Figure 5d, with isotype control antibody followed by secondary antibody conjugated to Alexa fluor 488. Shown is a cross-section of skin with isotype control antibody signals in green, collagen VII in red, bacteria in orange and nuclei in blue. Uninfected EVOCs were included as controls. DIC is included in the left panel image. Images were acquired at 20X and 100X objective magnification and the scalebars correspond to 100µm and 20µm respectively. The figure was labelled according to the following legend – D: Dermis, E: Epidermis, SC: Stratum corneum, BM: Basement membrane, DE: Detached epidermis.

**Supp Video 1.** 3D rendering sites of damage in 3D skin equivalent culture of GFP LifeAct expressing N/TERT-1 keratinocytes wounded with a needle and cultured with mApple labelled Bt*,* fixed at 0.67hpi (40min).

**Supp Video 2.** 3D rendering sites of damage in 3D skin equivalent culture of GFP LifeAct expressing N/TERT-1 keratinocytes wounded with a needle and cultured with mApple labelled Bt*,* fixed at 6hpi.

**Supp Video 3.** 3D rendering sites of damage in 3D skin equivalent culture of GFP LifeAct expressing N/TERT-1 keratinocytes wounded with a needle and cultured with mApple labelled Bt*,* fixed at 10hpi.

**Supp Video 4.** 3D rendering sites of damage in 3D skin equivalent culture of uninfected GFP LifeAct expressing N/TERT-1 keratinocytes wounded with a needle and cultured with mApple labelled Bt*,* fixed at 10hpi used as a control.

**Supp Video 5.** 2D scratch wound assay of N/TERT-1 keratinocytes infected with mApple labelled live Bt and uninfected cultures (control) from 5 to 24hpi.

**Supp Video 6.** Time-lapse imaging of Bt infection (red) of primary keratinocytes grown in monolayer showing extrusion of a single cell in 10fps.

**Supp Video 7.** Time-lapse imaging of Bt infection (red) of primary keratinocytes grown in monolayer showing extrusion of a group of cells in 10fps.

**Supp Video 8.** Confocal z-stack of N/TERT-1 keratinocytes expressing GFP LifeAct infected by mApple labelled Bt that has been extruded from the keratinocyte monolayer. Video of z-slices taken from top of infected cell to the epithelial monolayer (each frame is a z-slice, step size is 0.3µm). Scalebar = 50µm.

**Supp Video 9.** Sequence of events showing that Bt infected N/TERT-1 ASC-GFP expressing keratinocytes undergo ASC activation, cell death and subsequent cellular extrusion. Bt chromosomally express mApple fluorescent protein and are displayed in red. N/TERT-1 keratinocytes express ASC-GFP, which is displayed in green. Cell impermeant TO-PRO-3 nucleic acid stain was used to label the nuclei of dead cells and is displayed in blue.

**Supp Video 10.** Time-lapse imaging of Bt infection (orange) of N/TERT-1 ASC-GFP expressing keratinocytes showing that keratinocyte infection leads to ASC activation in neighboring cells (ASC specks were observed).
